# Actigraphy-based sleep outcomes in substance use disorders: A protocol for a systematic review and meta-analysis

**DOI:** 10.1371/journal.pone.0340595

**Published:** 2026-01-07

**Authors:** Alisson M. Paredes Naveda, Henrique Nunes Pereira Oliva, Delaram J. Ghadimi, Gustavo A. Angarita

**Affiliations:** 1 Department of Psychiatry, Yale University School of Medicine, New Haven, Connecticut, United State of America; 2 Connecticut Mental Health Center, New Haven, Connecticut, United States of America; 3 Southern Connecticut State University, New Haven, Connecticut, United State of America; 4 Clinical Neuroscience Research Unit, Connecticut Mental Health Center, New Haven, Connecticut, United States of America; 5 School of Medicine, Shahid Behesti University of Medical Sciences, Tehran, Iran; Portugal Football School, Portuguese Football Federation, PORTUGAL

## Abstract

**Introduction:**

Individuals with substance use disorders (SUDs) are at increased risk for sleep disturbances, creating a bidirectional relationship that may heighten relapse risk. While polysomnography is the gold standard for measuring sleep, many studies have used actigraphy, a noninvasive, wrist-worn device that estimates rest-activity patterns and sleep-wake characteristics. Despite its utility, the use of actigraphy in populations with SUDs remains limited, and findings vary across substances and methodologies. This protocol outlines a systematic review and meta-analysis aiming to synthesize evidence on actigraphy-derived sleep outcomes across various substances.

**Method and analysis:**

We will include peer-reviewed observational or interventional studies involving individuals aged 18 or older with a diagnosis of substance use (e.g., alcohol, benzodiazepines, cannabis, cocaine, opioids, methamphetamine, or nicotine) who use actigraphy to assess sleep compared to matched controls. Studies will be excluded during screening if they do not use actigraphy, do not include populations with SUDs, or focus on participants younger than 18. There will be no restrictions on location, setting, or language. Databases to be searched include PubMed, Scopus, Web of Science, ProQuest, ClinicalTrials.gov, CINAHL, PsycINFO, and Embase. Titles and abstracts will be screened in the first phase, followed by full-text screening using eligibility criteria. At least two independent reviewers will assess risk of bias using the Risk Of Bias In Non-randomized Studies of Exposures (ROBINS-E) tool. A synthesis will summarize key findings, including study characteristics, population differences, and methodological variations.

**Discussion:**

This review will offer a clear and comprehensive assessment of the current literature on actigraphy to examine sleep in SUD and to inform future research to study sleep and its implications in SUD populations.

**Protocol registration number:**

International Prospective Register for Systematic Reviews (PROSPERO) number CRD420251072028.

## Introduction

According to the World Drug Report 2023, in 2021, over 296 million people used substances worldwide [1]. Substance Use Disorders (SUDs) are characterized by patterns of substance use ranging from mild to severe, often taking significant toll on individuals’ physical and mental health. One risk is premature death, stemming from overdoses and long-lasting negative effects. These lasting changes in the brain can include alterations in the reward system, executive functioning, mood, self-awareness, and sleep [2]. Furthermore, analysis of the global prevalence of mental and substance use disorders in 2019 found that the worldwide prevalence of SUDs was 2.2%, with alcohol use disorder (AUD) being the most common at 1.5%, followed by other substances such as cannabis, opioids, amphetamine, and cocaine [3]. Among those affected, 13% experience psychiatric issues, with depression affecting about 5.7% [4]. In addition, sleep disturbances are also common among this population.

While there is no exact number of individuals with SUDs who experience sleep disturbances due to variability among substance types, research consistently shows that sleep problems are highly prevalent in this population, including difficulty falling asleep, difficulty staying asleep, impaired daytime functioning, and more [5]. For instance, a study done by Mahfoud et al. [6] indicated that self-reported sleep impairment – using the Pittsburgh Sleep Quality Index (PSQI) – was the primary issue among participants with AUD (80%), polysubstance (66%), and narcotics (40%). These findings indicate a high prevalence among this population compared to the general population, where only a small number of individuals are affected (1.5%). Furthermore, the most common sleep-related disorders are insomnia, circadian rhythm disorders, and sleep-related breathing disorders [5]. The bidirectional relationship between SUDs and sleep makes it difficult for treatment-seeking patients to adhere to treatment. Substance use leads to worse sleep outcomes, which increases the risk of relapse [7]. Moreover, during withdrawal, individuals can experience many symptoms such as cravings and poor sleep quality, and in an attempt to alleviate sleep disturbances, they come back to the use of the substance previously used [8,9].

In sleep studies, it is common to use both objective and subjective measures, each with its own importance. Subjective measures are often assessed using tools such as the PSQI; these measures rely on self-reported experiences, such as how restful the individual feels or how long they believe they sleep [10]. It is important to capture and understand the psychological or perceptual factors that influence behavior and can be beneficial when access to objective measures is unavailable. However, these measures are prone to biases (e.g., recall biases), and patients tend to under- or over-report the severity of their sleep disturbance [11]. On the contrary, objective measures such as polysomnography (PSG) can control for these variables and measure sleep more precisely. PSG is widely considered to be the gold standard in sleep studies due to being a multiparameter assessment that can assess brain activity by using electroencephalography, assess eye movement by using electrooculography, assess muscle activity by using electromyography, and cardiac activity by using electrocardiography [11,12]. Although it can be widely used, the use of PSG is typically assessed in laboratory settings. This can bring many disadvantages, such as burden, cost, and discomfort due to having to adapt to a new setting, affecting sleep [12].

Therefore, several studies have opted for the use of actigraphy in clinical and research settings [10]. Actigraphy is a noninvasive measure that assesses rest-activity patterns and estimates sleep-wake characteristics [13]. It is typically used via a wrist-worn device, on the non-dominant wrist or the ankle [13]. It is capable of measuring sleep parameters such as total sleep time (TST), sleep efficiency (SE), sleep latency (SL), and wake after sleep onset (WASO). In addition, wrist-worn devices bring many benefits to sleep assessments. For instance, it is cost-effective, and it can be used in real-world settings, such as home. Because it allows ecological monitoring, it is easier to accomplish longitudinal studies [12]. Moreover, actigraphy is clinically validated and recommended for assessing and supporting the diagnosis of several sleep-related conditions, such as insomnia and circadian rhythm sleep-wake disorders (CRSWDs). This is because it can estimate sleep patterns and variability over time, and can be beneficial when PSG is not feasible or too burdensome [10,11]. However, actigraphy cannot assess sleep architecture, as it cannot distinguish between sleep stages, lacking electroencephalogram, electrooculogram, and electromyogram data [10]. Even though Actigraphy itself can assess sleep parameters, it is usually recommended to use sleep logs, as they can help define rest intervals such as bedtime [14,15]. Actigraphy also requires an event marker, a button that the subject can press to mark several events (e.g., naps, drowsiness) [10]. While actigraphy can identify insomnia and CRSWDs [16,17], there are some variabilities that can affect its effectiveness [18–20].

For instance, actigraphy has a high sensitivity and accuracy similar to PSG, but low specificity, which makes it difficult to detect wakefulness within sleep periods [10,21]. Additionally, a variability of definitions and technical details (e.g., algorithms and sensitivity threshold settings) in wrist-worn devices was noted among studies [12]. For example, determining bedtime was not systematically defined in studies, as ‘bedtime’ was based on the sleep logs, while other studies based it on event markers [12]. Failure to report data was also common among studies; while some studies completely relied on actigraphy algorithms to estimate SL per instance, other studies failed to report these details [22–24]. Moreover, the selection of instruments, data processing, and analysis are not always described in studies [25]. Studies also failed to include circadian rhythm variables, which are crucial, as these variables track patterns of rest and activity over time, which reflect the individuals’ biological clock [25]. Lastly, a meta-analysis by Smith et al. [12] studied the use of actigraphy in sleep disorders and CRSWDs, and findings could not be generalized due to its small and heterogeneous sampling. Similarly, a systematic review found similar limitations in regard to sampling limitations [10].

Despite the increasing use of actigraphy in sleep research, a systematic review is needed to further analyze and summarize actigraphy-based sleep outcomes in individuals with SUDs. Due to the variabilities in the methodology, such as the type of device used or how sleep parameters are measured, as well as its heterogeneous sampling, it is difficult to compare among studies. This review aims to identify and describe sleep parameters derived from actigraphy across various substance types, including alcohol, benzodiazepines, cannabis, cocaine, opioids, methamphetamine, and nicotine. Methodological characteristics and study quality will be evaluated to assess what is currently in literature and what the limitations are. Lastly, this review seeks to address these limitations by comprehensively synthesizing previous research and providing a better understanding, and informing future research to come for later meta-analytical analyses, as well as the application in clinical settings. This will further help understand sleep alterations in populations with SUDs and how to better develop treatments suitable for each substance.

### Research questions

The purpose of this systematic review and meta-analysis is to answer the following research questions: 1) “What actigraphy-derived sleep parameters have been assessed in individuals with SUDs?”; 2) “How do actigraphy-based sleep outcomes vary across different types of substances?”; 3) “What are the methodological characteristics, strengths, and limitations of studies using actigraphy to assess sleep in individuals with SUDs?”; and 4) “What are the capabilities and limitations of wearable actigraphy devices in detecting and differentiating between sleep stages in individuals with SUDs?”

### Aims and objectives

The aim of this systematic review and meta-analysis is to identify and synthesize the existing evidence on sleep outcomes measured by actigraphy in individuals with SUDs. Considering the growing recognition of sleep disturbances as a clinically significant issue in this population, the review will focus on the use of actigraphy for assessing sleep-wake patterns in naturalistic settings.

The specific objectives of the review are to:

- Identify and describe actigraphy-derived sleep parameters in individuals with SUDs, including:
  a) Total sleep time (TST)
  b) Sleep efficiency (SE)
  c) Sleep latency (SL)
  d) Wake after sleep onset (WASO)
- Examine differences in sleep outcomes by substance type, including:
  a) Alcohol
  b) Benzodiazepines
  c) Cannabis
  d) Cocaine
  e) Opioids
  f) Methamphetamine
  g) Nicotine
- Analyze the methodological and reporting quality of studies (e.g., device type variation, scoring methods, study design)
- Identify gaps and limitations in literature and provide recommendations to guide future research and clinical applications of actigraphy in SUDs context.

## Materials and methods

The Preferred Reporting Items for Systematic Reviews and Meta-Analyses Protocols (PRISMA-P) 2015 checklist [26] was used to write this protocol. It can be found in S2 Table. The protocol was registered with the International Prospective Register of Systematic Reviews (PROSPERO) on July 2, 2025. The registration number is CRD420251072028 [27]. The protocol contains information such as eligibility criteria, selected databases, data collection, and the risk of bias assessment. For the full systematic review, the PRISMA 2020 checklist [28] will be used when writing to assess a clear and complete report.

### Eligibility criteria

#### Inclusion criteria.

Included studies must fit the following criteria:

- Studies including adults ages 18 and older with a current or past diagnosis of substance use disorder.
- Studies should include a matched comparison group, in which subjects are not exposed to substances.
- Psychiatric comorbidities and/or treatment exposure will be considered, provided the comparison group can be matched for these variables.
- Objective sleep measures should be assessed using actigraphy. Studies can also include self-report or clinician-rated sleep measures, but actigraphy must be a primary tool used to assess sleep parameters.
- Observational or interventional studies.

#### Exclusion criteria.

The following studies will be excluded:

- Studies that include only samples from participants younger than 18 years.
- Studies that include only SUD populations without a matched comparison group.
- Studies in which the focus is exclusively on non-SUD populations.
- Studies that did not include objective sleep data collected via actigraphy.
- Review articles, meta-analyses, qualitative studies, editorials, and commentaries.
- Case reports, conference abstracts, and proceedings.

### Databases to be searched

The following electronic databases will be searched:

- PubMed, Scopus, Web of Science (Core Collection), ProQuest (Databases), Clinicaltrials.gov, CINAHL (EBSCO), PsycINFO, and Embase (Elsevier).

Reference lists of included studies will be screened for additional eligible publications.

### Search strategy

A comprehensive search strategy was developed and tested on PubMed on June 12, 2025. No restriction will be placed on language or publication year. Articles published in languages other than English will be translated if needed. Any amendments to the search strategy or inclusion/exclusion criteria during the review process will be transparently documented in the final manuscript. See S1 Table for keywords structured under the PICOS (Population, Intervention, Comparison, Outcome, Study Design) framework [29] used in the search strategy for PubMed.

### Study records

#### Data management.

The file from each database will be imported by the first author (A.M.P.N) into Covidence, a systematic review management platform. Duplicates will be identified and removed.

### Study selection

Two independent review authors (A.M.P.N. and H.N.P.O.) will select in two phases. In Phase 1, they will screen titles and abstracts according to the eligibility criteria. In Phase 2, the same reviewers will independently assess the full texts of potentially eligible studies to determine final inclusion.

Discrepancies at any stage will be resolved through discussion. If agreement cannot be reached, a third reviewer (G.A.A) will be consulted to make the final decision. Moreover, if data is missing or unclear, attempts will be made to contact the study’s corresponding author for clarification. All studies excluded at the full-text stage, along with the reasons for exclusion, will be documented and presented in an appendix in the final review.

### Data collection and extraction

Data will be extracted independently by two reviewers. A standardized data extraction form will be developed in Microsoft Excel. One reviewer (A.M.P.N.) will perform the initial extraction, and a second reviewer (H.N.P.O.) will verify the extracted information. Discrepancies will be resolved through discussion, and a third reviewer (G.A.A) will be consulted if consensus cannot be reached. The collected information will include the author(s), year of publication, country, study design, sample size, participant demographics (e.g., age, sex), type of substance use disorder (e.g., alcohol use disorder), and sleep measure parameters (e.g., total sleep time). Subjective sleep measures (e.g., PSQI) will be compare with actigraphy data. Outcomes will include findings related to sleep quality and quantity, differences by substance type, and any reported correlations with mental health. Moreover, methodological details such as the duration of actigraphy monitoring, scoring approach (e.g., diary-assisted), and issues related to compliance or data loss will also be collected. Study limitations, whether reported or identified by authors during the review process, will be recorded. Extracted data and analyses will be provided in the final publication and its supplemental materials. Additionally, all relevant data will be made available upon reasonable request.

### Risk of bias assessment

The risk of bias of the included studies will be assessed using the Risk of Bias In Non-Randomized Studies of Exposure (ROBINS-E) tool [30]. This tool evaluates the methodological quality of studies across key domains, including confounding bias, measurement of the exposure bias, selection of participant bias, post-exposure intervention bias, missing data bias, measurement of the outcome bias, and selection of the reported results bias. Moreover, risk of bias will be assessed independently by two reviewers (A.M.P.N and H.N.P.O.). Any discrepancies will be resolved through discussion, and if consensus cannot be reached, a third reviewer (G.A.A) will be consulted.

If required information is missing or unclear, attempts will be made to contact the study authors for clarification. Moreover, risk of bias assessments will be applied at the study level rather than the outcome level, and no study will be excluded solely due to its risk of bias. Summary figures will be created using the *robvis* visualization tool [31].

### Data synthesis

A narrative synthesis of the included studies will be made, summarizing study characteristics, population differences, substance type, actigraphy methodology, and key findings related to sleep parameters. Methodological variations, including scoring procedures, monitoring duration, and participant compliance, will be described to contextualize differences across studies. Evaluation of measurement properties and reporting quality will be informed by COnsensus-based Standards for the selection of health Measurement INstruments (COSMIN) [32] guidelines to ensure consistent appraisal of actigraphy methodology. Additional sensitivity analyses will be performed to evaluate the robustness of findings, excluding studies judged to be at high risk of bias based on the ROBINS-E assessment tool. If available, subjective sleep measures (e.g., PSQI) will also be reported and compared to actigraphy data.

The data analysis will be conducted using Review Manager (RevMan). We will compare sleep outcomes between individuals with SUDs and matched control groups. The outcomes to be included in our meta-analysis will be TST, SE, SL, and WASO. Random-effects models will be used to account for between-study variability, and pooled estimates will be calculated with 95% confidence intervals, and an alpha level of 0.05 for statistical significance. If substantial heterogeneity is observed, potential sources will be identified through sensitivity analyses. In particular, variability in outcome definitions will be harmonized or analyzed separately. Statistical heterogeneity will be assessed using the I^2^ statistic. Analyses conducted using RevMan may be complemented by using additional software (e.g., SPSS or R). Moreover, we will generate forest plots to visually summarize the effect sizes and their precision across the included studies.

### Analysis of subgroups

The results will be analyzed by subgroups if data is available to explore variations in sleep outcomes across specific groups. Planned analyses include population characteristics (e.g., age group, sex, clinical vs. community samples), type of substance, stage of SUD (e.g., withdrawal, early abstinence), and sleep assessment duration (e.g., short-term vs. long-term actigraphy monitoring). Subgroup analyses will be conducted if the number of articles identified in the systematic review is appropriate. Subgroup analyses will be conducted for the sex variable, level (e.g., acute, chronic), and the stage of SUD (e.g., early withdrawal, late withdrawal, relapse). These analyses will be important since they will help contextualize differences in sleep patterns across substance types and usage stages [33], as well as methodological variability [10].

### Assessment of certainty evidence

The certainty of evidence across included studies will be evaluated using the Grading of Recommendations Assessment, Development, and Evaluation (GRADE) approach [34]. GRADE assesses the overall quality of evidence for each outcome by examining five domains, such as risk of bias, inconsistency, indirectness, imprecision, and publication bias. Based on this evaluation, the certainty of evidence for each outcome will be rated as high, moderate, low, or very low. This assessment will inform the interpretation of findings and the strength of conclusions drawn from the review.

## Discussion

Sleep disturbances are highly prevalent among individuals with SUDs. Its bidirectional relationship is known to influence the development of the disorder, relapse risk, treatment outcomes, mental health outcomes, and overall quality of life. Although PSG is considered the gold standard in sleep research, newer findings show that actigraphy offers a more cost-effective and ecologically valid method for monitoring sleep patterns in natural settings over extended periods. However, the existing literature on actigraphy remains unclear, with significant variability in study design, substance type, monitoring duration, and analytic methods. This systematic review aims to consolidate the available evidence on objective sleep characteristics in individuals with SUDs, with the goals of identifying consistent patterns, methodological limitations, and gaps in knowledge.

Focusing on a non-invasive and cost-effective tool such as actigraphy in clinical and research contexts will help clarify differences between substance types in sleep outcomes. Ultimately, findings may allow the development of appropriate treatments, the identification of high-risk periods for relapse, and the provision of adequate care strategies for individuals with SUDs.

### Implications for research and practice

This review will employ a comprehensive search strategy across multiple databases, use a validated tool for risk of bias assessment, and follow the PRISMA-P 2015 guidelines to ensure transparency and methodological rigor. If the inclusion of both subjective and objective sleep measures is possible, it will allow for a more nuanced comparison of sleep experienced in this population. However, limitations may include heterogeneity, which may limit the feasibility of conducting a meta-analysis. Lastly, while no language restrictions will be applied, the availability of full texts or translations may affect inclusion.

### Timeline and status of the review

As of this submission, we have piloted the screening process to ensure the feasibility of our procedures, but full systematic screening and data extraction have not yet been completed. We anticipate initiating formal title and abstract screening in August, with full-text review completed by late September. Data extraction and quality assessment are planned for October. We aim to complete data synthesis and draft initial results by the end of November, with a final report ready by December 1, 2025, in accordance with our registered PROSPERO timeline.

### Dissemination plans

The systematic review will be submitted for publication in a peer-reviewed journal and presented to clinical and research audiences involved in treatments of SUDs. Findings will also be shared through academic conferences, webinars, and collaborative research networks to maximize impact and promote integration of sleep-focused measures in SUD care.

### Amendments to the study protocol

There are no planned amendments to this systematic review protocol at the time of writing. However, if any changes become necessary during the review process, they will be documented. Each amendment will include the date of the change, a description of the revision, and the rationale behind it. All amendments will be reflected in both the PROSPERO registration (if applicable) and the final published review to ensure transparency.

## Supporting information

S1 Table Search strategy. Keywords to be used in the search organized into blocks.
(DOCX)

S2 Table PRISMA-P 2015 checklist. Recommended items to address in a systematic review protocol.
(DOCX)

## References

[1] United Nations Office on Drugs and Crime. World Drug Report 2023. http://www.unodc.org/unodc/en/data-and-analysis/world-drug-report-2023.html. Accessed 2025 July 14.

[2] Volkow ND, Blanco C. Substance use disorders: a comprehensive update of classification, epidemiology, neurobiology, clinical aspects, treatment and prevention. World Psychiatry. 2023;22(2):203–29. doi: 10.1002/wps.21073 37159360 PMC10168177

[3] Castaldelli-Maia JM, Bhugra D. Analysis of global prevalence of mental and substance use disorders within countries: focus on sociodemographic characteristics and income levels. Int Rev Psychiatry. 2022;34(1):6–15. doi: 10.1080/09540261.2022.2040450 35584016

[4] Chavan BS, Garg R, Das S, Puri S, Banavaram AA. Prevalence of substance use disorders in Punjab: findings from national mental health survey. Indian J Med Res. 2019;149(4):489–96. doi: 10.4103/ijmr.IJMR_1267_17 31411172 PMC6676856

[5] Chakravorty S, Vandrey RG, He S, Stein MD. Sleep management among patients with substance use disorders. Med Clin North Am. 2018;102(4):733–43. doi: 10.1016/j.mcna.2018.02.012 29933826 PMC6289280

[6] Mahfoud Y, Talih F, Streem D, Budur K. Sleep disorders in substance abusers: how common are they?. Psychiatry (Edgmont). 2009;6(9):38–42. 19855859 PMC2766287

[7] Teeters JB, Jones JL, Jarnecke AM, Back SE. Sleep moderates the relationship between stress and craving in individuals with opioid use disorder. Exp Clin Psychopharmacol. 2021;29(4):418–26. doi: 10.1037/pha0000372 32297784 PMC8375668

[8] Cheatle MD, Webster LR. Opioid therapy and sleep disorders: risks and mitigation strategies. Pain Med. 2015;16 Suppl 1(0 1):S22-6. doi: 10.1111/pme.12910 26461072 PMC4608386

[9] Eacret D, Veasey SC, Blendy JA. Bidirectional relationship between opioids and disrupted sleep: putative mechanisms. Mol Pharmacol. 2020;98(4):445–53. doi: 10.1124/mol.119.119107 32198209 PMC7562980

[10] Liguori C, Mombelli S, Fernandes M, Zucconi M, Plazzi G, Ferini-Strambi L, et al. The evolving role of quantitative actigraphy in clinical sleep medicine. Sleep Med Rev. 2023;68:101762. doi: 10.1016/j.smrv.2023.101762 36773596

[11] Huhn AS, Dunn KE, Ellis JD, Sholler DJ, Tabaschek P, Burns R, et al. Objective sleep outcomes in randomized-controlled trials in persons with substance use disorders: a systematic review. Drug Alcohol Depend. 2022;237:109509. doi: 10.1016/j.drugalcdep.2022.109509 35660222

[12] Smith MT, McCrae CS, Cheung J, Martin JL, Harrod CG, Heald JL, et al. Use of actigraphy for the evaluation of sleep disorders and circadian rhythm sleep-wake disorders: an American academy of sleep medicine systematic review, meta-analysis, and GRADE assessment. J Clin Sleep Med. 2018;14(7):1209–30. doi: 10.5664/jcsm.7228 29991438 PMC6040804

[13] Hasler BP, Schulz CT, Pedersen SL. Sleep-related predictors of risk for alcohol use and related problems in adolescents and young adults. Alcohol Res. 2024;44(1):02. doi: 10.35946/arcr.v44.1.02 38500552 PMC10948113

[14] Jang KH, Lee JH, Kim SJ, Kwon HJ. Characteristics of napping in community-dwelling insomnia patients. Sleep Med. 2018;45:49–54. 29680428 10.1016/j.sleep.2017.12.018

[15] Kay DB, Dzierzewski JM, Rowe M, McCrae CS. Greater night-to-night variability in sleep discrepancy among older adults with a sleep complaint compared to noncomplaining older adults. Behav Sleep Med. 2013;11(2):76–90. doi: 10.1080/15402002.2011.602775 23137288

[16] Rajna P, Takács J. Diagnosis of primary insomnia by actigraphy--improved results by data selection. Ideggyogy Sz. 2014;67(1–2):43–51. 24654446

[17] Natale V, Léger D, Martoni M, Bayon V, Erbacci A. The role of actigraphy in the assessment of primary insomnia: a retrospective study. Sleep Med. 2014;15(1):111–5. doi: 10.1016/j.sleep.2013.08.792 24325809

[18] Levenson JC, Troxel WM, Begley A, Hall M, Germain A, Monk TH. A quantitative approach to distinguishing older adults with insomnia from good sleeper controls. JCSM. 2013;9(2):125–31. 23372464 10.5664/jcsm.2404 PMC3544379

[19] Bottary R, Seo J, Daffre C, Gazecki S, Moore KN, Kopotiyenko K, et al. Fear extinction memory is negatively associated with REM sleep in insomnia disorder. Sleep. 2020;43(7):zsaa007. doi: 10.1093/sleep/zsaa007 31993652 PMC7355402

[20] Devine JK, Bertisch SM, Yang H, Scott-Sutherland J, Wilkins A, Molina V, et al. Glucocorticoid and inflammatory reactivity to a repeated physiological stressor in insomnia disorder. Neurobiol Sleep Circadian Rhythms. 2018;6:77–84. doi: 10.1016/j.nbscr.2018.06.001 31236523 PMC6586925

[21] Angelova M, Karmakar C, Zhu Y, Drummond SPA, Ellis J. Automated method for detecting acute insomnia using multi-night actigraphy data. IEEE Access. 2020;8:74413–22. doi: 10.1109/access.2020.2988722

[22] Blackwell T, Ancoli-Israel S, Redline S, Stone KL. Factors that may influence the classification of sleep-wake by wrist actigraphy: the MrOS sleep study. J Clin Sleep Med. 2011;7(4):357–67. 21897772 10.5664/JCSM.1190 PMC3161768

[23] Adam T, Tanty J, Barateau L, Dauvilliers Y. Actigraphy against 32-hour polysomnography in patients with suspected idiopathic hypersomnia. J Sleep Res. 2025;34(5):e70007. doi: 10.1111/jsr.70007 39979124 PMC12426704

[24] Palotti J, Mall R, Aupetit M, Rueschman M, Singh M, Sathyanarayana A, et al. Benchmark on a large cohort for sleep-wake classification with machine learning techniques. NPJ Digit Med. 2019;2:50. doi: 10.1038/s41746-019-0126-9 31304396 PMC6555808

[25] Berger AM, Wielgus KK, Young-McCaughan S, Fischer P, Farr L, Lee KA. Methodological challenges when using actigraphy in research. J Pain Symptom Manage. 2008;36(2):191–9. doi: 10.1016/j.jpainsymman.2007.10.008 18400460 PMC2542506

[26] Shamseer L, Moher D, Clarke M, Ghersi D, Liberati A, Petticrew M, et al. Preferred reporting items for systematic review and meta-analysis protocols (PRISMA-P) 2015: elaboration and explanation. BMJ. 2015;350:g7647. doi: 10.1136/bmj.g7647 25555855

[27] Paredes NavedaA, OlivaH, Jafarzadeh GhadimiG, AngaritaG. Actigraphy-Based Sleep Outcomes in Substance Use Disorders: A Systematic Review. PROSPERO. 2025. https://www.crd.york.ac.uk/PROSPERO/view/CRD42025107202810.1371/journal.pone.034059541499544

[28] Page MJ, McKenzie JE, Bossuyt PM, Boutron I, Hoffmann TC, Mulrow CD, et al. The PRISMA 2020 statement: an updated guideline for reporting systematic reviews. BMJ. 2021;372:n71. 10.1136/bmj.n71 PMC8005924 33782057

[29] Methley AM, Campbell S, Chew-Graham C, McNally R, Cheraghi-Sohi S. PICO, PICOS and SPIDER: a comparison study of specificity and sensitivity in three search tools for qualitative systematic reviews. BMC Health Serv Res. 2014;14:579. doi: 10.1186/s12913-014-0579-0 25413154 PMC4310146

[30] Higgins JPT, Morgan RL, Rooney AA, Taylor KW, Thayer KA, Silva RA, et al. A tool to assess risk of bias in non-randomized follow-up studies of exposure effects (ROBINS-E). Environ Int. 2024;186:108602. doi: 10.1016/j.envint.2024.108602 38555664 PMC11098530

[31] McGuinness LA, Higgins JPT. Risk-of-bias VISualization (robvis): An R package and Shiny web app for visualizing risk-of-bias assessments. Res Synth Methods. 2021;12(1):55–61. doi: 10.1002/jrsm.1411 32336025

[32] Mokkink LB, Terwee CB, Knol DL, Stratford PW, Alonso J, Patrick DL, et al. Protocol of the COSMIN study: Consensus-based standards for the selection of health measurement instruments. BMC Med Res Methodol. 2006;6:2. doi: 10.1186/1471-2288-6-2 16433905 PMC1368990

[33] Gordon HW. Differential effects of addictive drugs on sleep and sleep stages. J Addict Res (OPAST Group). 2019;3(2):10.33140/JAR.03.02.01. doi: 10.33140/JAR.03.02.01 31403110 PMC6688758

[34] Guyatt GH, Oxman AD, Vist GE, Kunz R, Falck-Ytter Y, Alonso-Coello P, et al. GRADE: an emerging consensus on rating quality of evidence and strength of recommendations. BMJ. 2008;336(7650):924–6. doi: 10.1136/bmj.39489.470347.AD 18436948 PMC2335261

