# Supplementary material for: Actigraphy-based sleep outcomes in substance use disorders: A protocol for a systematic review and meta-analysis
Source: PLoS One. 2026 Jan 7;21(1):e0340595. doi: 10.1371/journal.pone.0340595 (PMC12779124; doi:10.1371/journal.pone.0340595)
Supplement: S1 Table — Keywords to be used in the search organized into blocks. (DOCX) [file pone.0340595.s001.docx]

**Supporting Table 1**

### **S1 Table. Search strategy.** Keywords to be used in the search organized into blocks

| **Blocks (PICOS)** | **Keywords** |
| --- | --- |
| **P**(Population) | “Substance Use Disorder” OR “Substance-Related Disorders” OR “Drug Addiction” OR “Drug Addict” OR “Drug Dependence” OR “Substance Abuse” OR “Substance Use” OR “Addiction” OR “Addictive Behavior” OR “Withdrawal” OR “Substance Withdrawal Syndrome” OR “Drug Abuse” OR “Drug Dependency” OR “Drug Use” OR “Drug Related Disorder” OR “Drug Related Disorders” OR “Substance Dependence” OR “Substance Dependency” OR “Substance Addiction” OR “Alcohol Use Disorder” OR “Alcoholism” OR “Alcohol Abuse” OR “Alcohol Dependence” OR “Alcohol Dependency” OR “Alcohol Addiction” OR “Alcohol Related Disorder” OR “Alcohol Related Disorders” OR “Binge Drinking” OR alcohol OR “Opioid Use Disorder” OR “Opioid-Related Disorders” OR “Opioid Abuse” OR “Opioid Dependence” OR “Opioid Dependency” OR “Opioid Addiction” OR “Opioid Use” OR “Opioid Related Disorder” OR “Opioid Related Disorders” OR heroin OR morphine OR codeine OR oxycodone OR fentanyl OR opioids OR “Nicotine Use Disorder” OR “Tobacco Use Disorder” OR nicotine OR tobacco OR cigarettes OR “Cannabis Use Disorder” OR “Marijuana Abuse” OR “Marijuana Dependence” OR “Marijuana Addiction” OR cannabis OR marijuana OR weed OR “Cocaine Use Disorder” OR “Cocaine-Related Disorders” OR “Cocaine Abuse” OR “Cocaine Dependence” OR “Cocaine Dependency” OR “Cocaine Addiction” OR “Cocaine Use” OR “Cocaine Related Disorder” OR “Cocaine Related Disorders” OR cocaine OR crack OR “Benzodiazepine Use Disorder” OR benzodiazepines OR diazepam OR alprazolam OR xanax OR clonazepam OR “Methamphetamine Use Disorder” OR “Amphetamine-Related Disorders” OR “Amphetamine Abuse” OR “Amphetamine Dependence” OR “Amphetamine Dependency” OR “Amphetamine Addiction” OR “Amphetamine Use” OR “Amphetamine Related Disorder” OR “Amphetamine Related Disorders” OR methamphetamine OR “crystal meth” OR amphetamine OR “Injection Drug Use” |
| **I**(Intervention / Measurement) | “Actigraphy” OR “Actimetry” OR “Actigraph” OR “Wrist Actigraphy” OR “Wrist-worn Activity Monitor” OR “Wearable Sleep Tracker” OR “Wearable Activity Monitor” OR “Sleep Tracker” OR “Fitness Tracker” OR “Wrist-worn device” OR “Wearable Device” OR “Wearable Technology” OR “Accelerometry” OR “Activity Monitoring” OR “Motion Sensor” OR “Sleep Watch” OR “Index Sleep Monitor” |
| **C**(Comparison) | Not applicable (observational or descriptive study design expected) |
| **O** (Outcome) | “Sleep” OR “Sleep Initiation and Maintenance Disorders” OR “Sleep-Wake Transition Disorders” OR “Circadian Rhythm” OR “Circadian Rhythms” OR “Circadian Disruption” OR “Circadian Misalignment” OR “Sleep Disturbance” OR “Sleep Disturbances” OR “Sleep Problems” OR “Sleep Disorder” OR “Sleep Disorders” OR “Sleep Disruption” OR “Sleep Parameters” OR “Total Sleep Time” OR “Sleep Efficiency” OR “Sleep Latency” OR “Sleep Onset Latency” OR “Wake After Sleep Onset” OR “Sleep Fragmentation” OR “Sleep Quality” OR “Sleep Duration” OR “Rest-Activity Cycle” OR “Sleep-Wake Cycle” OR sleep OR insomnia |
| **S** (Study Design) | Optional: “Randomized controlled trial” OR “clinical trial” OR “observational study” OR “cross-sectional” OR “case-control” OR “cohort” (if filtering by design) |
